# Risk factors of adolescent exposure to violence in Burkina Faso

**DOI:** 10.1186/s12889-022-14854-7

**Published:** 2022-12-21

**Authors:** Ronald Musizvingoza, Nyasha Tirivayi, Frank Otchere, Francesca Viola

**Affiliations:** 1 United Nations University International Institute for Global Health (UNU IIGH), 56000 Kuala Lumpur, Malaysia; 2 UNICEF-Office of Research-Innocenti, Florence, Italy, Via Degli Alfani 58, 50121 Florence, Italy

**Keywords:** Children, Adolescence, Violence, Gender, Risk factors, Burkina Faso

## Abstract

**Background:**

Globally violence against children and adolescents is a significant public health problem. Since children rely on family for early learning and socialization, evidence of the factors associated with exposure to violence within households may inform the development of policies and measures to prevent violence and aid the victims of violence. This study examines the risk and protective factors associated with adolescents’ exposure to violence at home and how these differ by gender and age in four regions of Burkina Faso.

**Materials and methods:**

We used data from the baseline survey of the Child-Sensitive Social Protection Programme (CSSPP) conducted in four regions of Burkina Faso. The CSSPP is a cash transfer programme accompanied by complimentary nutrition, and water and sanitation interventions to address multidimensional child poverty. We employed bivariate and multivariable regression analysis on a sample of 2222 adolescents aged 10–19 to explore the risk and protective factors associated with exposure to violence.

**Results:**

Results show that exposure to psychological violence (22.7%) was more common within the households when compared to physical violence (9.1%). Adolescent girls reported more exposure to physical violence while boys reported more exposure to psychological violence. Significant risk factors associated with the likelihood of exposure to violence among girls are orphanhood, living in a household receiving safety nets and living in a Muslim-majority community. Among boys, age, school attendance, disability, a household receiving safety nets, sharing a household with a depressed individual, and living in a Muslim-majority community, were associated with exposure to violence.

**Conclusions:**

These gender-specific findings highlight the importance of family background characteristics and can be used to inform and strengthen the targeting of vulnerable children and adolescents in interventions aimed at reducing exposure to violence against children.

**Supplementary Information:**

The online version contains supplementary material available at 10.1186/s12889-022-14854-7.

## Background

Violence against children (VAC) and adolescents is a global public health and human rights problem. VAC includes physical, sexual, and psychological violence perpetrated against children and adolescents within and outside the family, as well as witnessing violence perpetrated against people below the age of 18 by parents, caregivers, peers, or strangers [1]. Globally, an estimated 1 billion children and adolescents between 2–17 years of age experience violence or neglect every year [2]. Evidence suggests that adolescents and children are more likely to experience certain forms of violence at different ages [3]. Almost 300 million (3 in 4) children aged between 2–4 years, experience violent discipline, while 250 million regularly suffer physical punishment from their caregivers (4). Worldwide, one-third of adolescent girls aged 15–19 have experienced physical and/or sexual violence by their partner [4]. Globally, about 200, 000 homicides occur each year among young people aged 10–29 years, while many more suffer from life-threatening injuries [5]. Homicide is among the top four leading causes of death in adolescents, with boys comprising over 80% of victims and perpetrators [5]. While violence against children and adolescents is common throughout the world, it is highest in Africa, Asia, and North America where at least 50% of children experienced violence in the past year [6]. When children and adolescents are exposed to violence, they suffer negative lifelong impacts on their health and well-being such as psychological harm, risky behaviours, poor health outcomes, educational outcomes, and involvement in crime [5]. However, evidence has shown that violence against children and adolescents can be prevented, and its impact reduced [7]. The Sustainable Development Goals, target 16.2 calls upon countries to “end abuse, exploitation, trafficking and all forms of violence and torture against children” [8]. Furthermore, international human rights treaties including the Convention on the Rights of the Child (CRC) enshrined the right of children to be protected from all forms of violence [9].

Research in low- and middle-income countries (LMICs) shows that the risk factors for violence against children extend beyond the characteristics of the children involved [10–12]. The social-ecological model explains the interplay between risk factors of violence at four levels: the individual, the relationship, the community, and the societal [13], which demonstrates the need for multi-level and multi-dimensional efforts that account for this complexity. A review of studies on VAC in Africa identified several individual, family and community-level risk and protective factors for physical and emotional violence [10]. Individual-level factors associated with violence against children and adolescents include age, disability, sex, exposure to bullying and a history of exposure to violence [11, 14, 15]. Risk factors at the household level include poverty, household violence, non-nuclear family, absence of biological fathers, closeness to mother and household socio-economic status [11, 14–19]. At the community level, food security, caring teachers at school and trusted community members are protective factors against violence among children [10, 11]. When exposed to violence, children experience both immediate and long-term negative health and social impacts [7]. Evidence from LMICs shows that exposure to violence increases risky sexual behaviours and the likelihood of contracting sexually transmitted diseases including HIV [20, 21]. Children and adolescents experiencing violence suffer from mental health problems, chronic diseases, reproductive health problems, and communicable and non-communicable diseases [20, 21]. Exposure to violence and adverse events is also linked to aggression, violence perpetration, substance use and suicide ideation among children and young people [22–25]. Among adolescent girls, exposure to gender-based violence has been linked with early pregnancy, female genital mutilation, and child marriages [21, 26].

In Burkina Faso, as in many settings, violence against children is a growing concern due to the deteriorating security situation emanating from violent extremism and conflict within the country and in the broader Sahel region. Women and girls are particularly vulnerable to internal displacements thereby becoming the primary victims of extreme violence such as rape, sexual exploitation, and child marriage [27]. Furthermore, extreme poverty among children in Burkina Faso results in a higher risk of violence, deprivation, and stress [28]. A national study showed that 16% and 26% of adolescents aged 12–17 years experienced physical and emotional violence respectively [29]. Additionally, children and children’s experience of violence differs across regions and rural–urban areas in Burkina Faso. For instance, the prevalence of physical violence was 53% in Mouhoun and 0.4% in Oudalan and Yagha among 12–17-year-olds [29]. While children are at risk of experiencing violence in many places, in Burkina Faso most children experienced violence at home, followed by school and in the streets [30].

Evidence on the magnitude, form, and predictors of violence against male and female children and adolescents during conflicts is sparse [31] and not fully understood [32, 33]. Moreover, assessing the prevalence and predictors of VAC and adolescents in fragile and conflict-affected settings such as Burkina Faso is a challenge as they lack the necessary reporting infrastructure [33]. To the authors’ knowledge, there is only one relevant previous study in Burkina Faso which examined how social expectations that favour the use of violence in education influence the experience of VAC in Burkina Faso [28]. Given the emergence of COVID-19 combined with armed conflict and climate shocks (drought) that result in internal displacement in Burkina Faso, there is a need for more evidence on the multi-level factors associated with violence against children in such a fragile and extremely poor context. This is especially important given the severe consequences of VAC in Burkina Faso which include low self-esteem, depression, and trauma among children [30]. Using data from the baseline survey of the Child-Sensitive Social Protection Programme (CSSPP) a Burkina Faso social protection programme, collected in regions facing multiple vulnerabilities that include multidimensional poverty, conflict and migration, we seek to close the gap on VAC in such settings. Therefore, this study will expand the existing evidence in Burkina Faso and contribute to the existing literature on VAC and adolescents overall, by exploring the relationship between household and community characteristics and exposure to violence against children and adolescents in fragile and conflict-affected settings.

## Material and methods

### Study design and setting

The study was conducted in eight municipalities in 4 regions of Burkina Faso namely: Boucle Du Mouhoun, East, North and Centre-North. Data used in this study came from the baseline survey which was part of the Child-Sensitive Social Protection Programme (CSSPP). The CSSPP is a social protection programme that combines cash, nutrition, water, sanitation, and hygiene (WASH) interventions to address multidimensional child poverty in selected regions in Burkina Faso. The four regions in this study were selected because of the high level of multidimensional poverty, estimated at 80% compared to the national average of 62% [34]. Furthermore, the regions experience an influx of transit migrants and refugees due to the ongoing conflict in the country. The regions are negatively affected by multiple vulnerabilities and receive limited investment in social sectors, factors which may erode community resilience, making women and children even more vulnerable to violence.

### Sampling and sample size

Households and respondents in this study were selected through a multistage sampling approach. Households were selected from a list of eligible households for the cash transfer intervention provided by the Le Secrétariat permanent du Conseil national pour la protection sociale (SP-CNPS). The first stage in the development of the sampling frame was the self-registration of vulnerable households by a household representative at the municipal social service. After self-registration “eligible” or “non‐eligible” beneficiary households were classified using an algorithm on household welfare based on households’ information and individual characteristics. The households included in the study were all determined to be eligible for the cash transfer under the CSSPP based on a proxy-means test (PMT) score. Households above the PMT score were excluded from the study. Additionally, a community validation exercise on the lists of identified vulnerable households was carried out.

The sample size for the study was determined based on power calculations to detect a reduction in poverty by 10 percentage points, with a power of 85%, a margin of error of 5% and a response rate of 90% between the baseline and endline study. Due to the use of multistage sampling, a design effect of 1.5 was used based on the design effect from the Demographic and Health Survey in Burkina Faso which used a similar multi-stage approach for sampling. The resulting sample size was 2,800 out of which 2,772 households were successfully interviewed. The head and one randomly selected adolescent between the ages of 10–19 in each household responded to the survey.

### Ethical considerations

The research protocol was reviewed and approved by the Ethical Review Committee of the Centre of Health Research of the Ministry of Health in Burkina Faso (2019–023-/MS/SG/INSP/CRSN/CIE). All participants provided written informed consent before participating in the survey. For participants below the age of 16, informed consent was obtained from a parent and/or legal guardian while verbal consent was provided by the participants.). The methods and procedures, including recruitment of participants, data collection, and analysis, were performed in accordance with the relevant guidelines and regulations from the ethical review board.

### Data collection

**Fig. 1:**
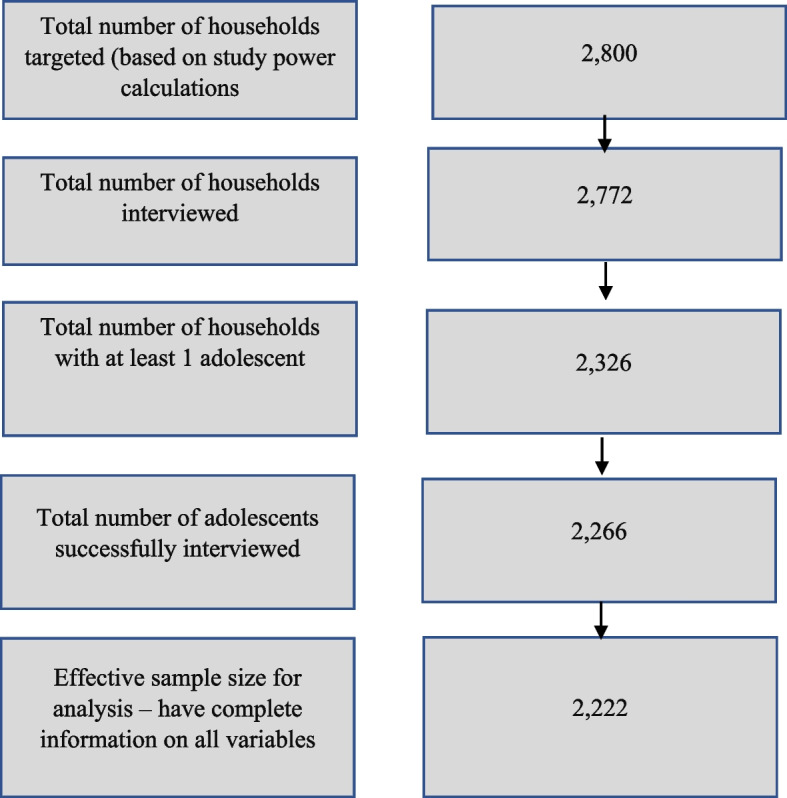
Schematic representation of the sampling process

Data were collected using three research instruments: a household, community, and adolescent questionnaire. A household questionnaire was administered to the household head and a community leader was randomly selected to respond to the community questionnaire. The adolescent questionnaire was administered to one randomly selected adolescent between the ages of 10–19 in each household where there was an adolescent. The questionnaire contains information on children's exposure to childhood adverse experiences including violence as well as their household and community living conditions. The outcome measure in this study, namely physical and emotional violence, is drawn from the adolescent questionnaire, whereas the explanatory variables were drawn from the adolescent, household, and community questionnaires. Out of the 2,772 households interviewed, there were 2,326 with at least one adolescent and 2,266 randomly selected adolescents were successfully interviewed. The effective sample for analysis was however 2,222 due to missing data on the dependent or independent variables for 44 adolescents. Figure 1 provides a summary of the sampling tree.

## Measures

### Physical and emotional violence

The outcome variable in this study is exposure to household violence in the 12 months preceding the survey. The study also examines two forms of violence, i.e., physical, and psychological violence, using internationally recognised definitions. The first outcome variable, exposure to physical violence in the household, was measured by asking respondents the following question “During the past 12 months, have you (or someone in your family) experienced physical violence?”.^1^ The second outcome variable, exposure to psychological violence, was measured by asking respondents whether they had experienced any of the following: insults, yelling, intimidation, and humiliation. Finally, an aggregate outcome variable was created to measure exposure to any form of violence in the household based on an adolescent having experienced or witnessed any physical and/or psychological violence in the previous 12 months. Our outcome variables are binary coded 1 if an adolescent was exposed to violence (physical, psychological, and any form of violence) and 0 otherwise.

### Risk and protective factors

Respondents in the survey were asked several questions on factors hypothesised to put children at risk of emotional or physical violence. These factors include household access to safety nets, the experience of food shocks, alcohol consumption and the presence of depressed individuals. The following community-level factors were also included: living in a community of members taking advantage of each other; living in a Muslim-majority community and mobile network coverage in the community. Table 1 presents the definitions of the risk and protective factors included in the analysis. Following the socio-ecological framework, the following socio-demographic variables were included in the analysis: age, household size, disability status, school attendance, orphanhood status and household wealth [13]. Household wealth status was measured using an asset index^2^ variable composed of five quintiles; a composite indicator created using principal component analysis [35].

**Table 1:** Definitions of risk factor variables examined in the analysis

Variable Name	Coding
**Age group**	10–14 years = 0; 15–19 years = 1
**School Attendance**	Not attending school = 0; Attending school = 1
**Disability Status**	Does not have a functional disability = 0; Has functional disability = 1
**Orphanhood Status**	Not an orphan = 0; Orphan = 1
**Household Size**	Up to 6 members (0–6) = 0; More than 6 members (> 6) = 1
**Household Wealth Quintile**	Wealth indices are created by combining household-level measures for assets and amenities using principal components analysis following Demographic and Health Survey (DHS) methodology (31)
**The household has Access to Safety Nets**^**a**^	No access to safety net = 0; Access to safety nets = 1
**The Household has inadequate food stock**^**b**^	Household does not have adequate food stock = 0, Household inadequate food stock = 1
**The household has a depressed member**	No depressed member = 0; Has depressed member = 1
**Household with alcohol consumption**	No alcohol consumption = 0; Alcohol consumption = 1
**Community members take advantage of each other**	People in the community do not take advantage of each other = 0; People in the community take advantage of each other = 1
**The community has a Muslim majority**	Majority religion is not Muslim = 0 = 0; Muslim is the majority religion = 1
**The community has a mobile Network**	No mobile network = 0; Mobile network available = 1

^a^This variable was constrcuted from a questions asking if households received any form of social saftey net in the past 12 months. Social safety nets included Free maize, Free food (other than maize), Food/Cash-for, Work Programme, School Feeding Programme,"Free distribution of likuni phala to children and mothers (Targeted Nutrition Programme)","Supplementary feeding for malnourished children at a nutritional rehabilitation unit", Scholarships/Bursaries for secondary education, Social Cash Transfer Programme, Direct cash transfers from others (development partners, NGOs), Community Based Childcare (CBCC), Vouchers or coupons to buy fertilizers or seeds, Village Savings & Loans Program, Other (Specify) _____________________________________.

^b^Situations considered in this variable are, Inadequate household food stocks due to small land size, Inadequate household food stocks due to lack of farm inputs, "Inadequate household food stocks due to lack of farm, Tools/drought animals, plough etc.", Not able to reach the market due to high transportation costs, Market very far from the village and No food in the market.

### Statistical analysis

Data were analyzed using Stata 14. The data was cleaned and sorted out first to check for consistency and missing information. First, frequency distributions including summary statistics were used to describe the characteristics of the respondents. We then used cross-tabulations and Pearson’s chi-squared (χ2) tests to examine associations between VAC and the explanatory variables for girls and boys. Finally, the study used multivariable logistic regression models to estimate the odds ratios for exposure to violence. These models included individual, household, and community background characteristics of the child. Three logistic models were fit for each of the three outcome variables of violence (physical, psychological and any violence). For each outcome, three separate models were fitted. Model 1 for all children, model 2 for boys and model 3 for girls. Results are presented as odds ratios and confidence intervals. In all regressions, standard errors are clustered by village level to account for intra-village correlation. A goodness-of-fit test was carried out after each regression, and we also tested for multicollinearity by analysing the variance inflation factor (VIF) of explanatory variables (available in the Appendix). For all inferential statistical analyses, the minimum threshold for statistical significance was set at *p* < 0.05.

## Results

### Sample characteristics

Table 2 presents the socio-demographic characteristics of the study population. The mean age of the adolescents was 13.6 years (SD:2.6). The sample was evenly distributed between males and females (50.3% and 49.7% respectively). School attendance was moderately high with over half (55.9%) of the children attending school. For girls, 42.0% were not in school and 36.7% were 15–19 years. Functional disability was low (1.0%), with boys (1.3%) twice as much as girls (0.6%) having at least one form of functional difficulty. Most children were living in households with 7 or more members (73%) and still had both parents alive (85.2%). For the outcome variable, 22.7% of the children were exposed to any form of violence in the past 12 months. More boys (23.7%) than girls (21.8%) were exposed to household violence. Exposure to psychological violence (20.1%) was more common when compared to physical violence (9.1%). More girls than boys reported exposure to physical violence (9.2% vs 8.9%) while the opposite was true for psychological violence (18.5.% vs 21.7%).

**Table 2:** Sample characteristics of the study population., (*N* = 2222)

Variable	Male	Female	Total
**Physical Violence**	8.9	9.2	9.1
**Psychological Violence**	21.7	18.5	20.1
**Any Violence**	23.7	21.7	22..7
**Mean Age (SD)**	13.5(2.6)	13.7(2.6)	13.6(2.6)
**Age Group**			
10–14	65.2	63.3	64.3
15–19	34.8	36.7	35.7
**School Attendance**			
Yes	48.4	51.6	55.9
No	46.2	42.0	44.1
**Disability Status**			
Yes	1.3	0.6	0.9
No	98.8	99.4	99.1
**Orphanhood Status**			
Not Orphan	85.0	85.4	85.2
Orphan	15.0	14.6	14.8
**Household Size**			
0–6	24.8	29.3	27.0
> 6	75.2	70.8	73.0
**Wealth Quintile**			
First quintile	17.8	20.0	18.9
Second quintile	20.2	20.1	20.1
Third quintile	21.5	19.8	20.6
Fourth quintile	20.8	19.5	20.1
Fifth quintile	19.7	20.6	20.1
**District**			
Boucle Du Mouhoun	23.0	18.8	20.9
East	16.9	19.7	18.3
North	30.6	33.7	32.1
Centre-North	29.5	27.9	28.7
**Total**	**1118(50.3)**	**1104(49.7)**	**2222(100.0)**

Table 3 presents the results of the bivariate analysis showing the association between each background characteristic and exposure to any violence, physical and psychological violence among children in Burkina Faso. The results show gender differentials in exposure to violence among boys and girls. For instance, girls reported a higher prevalence of physical violence (boys: 9.2% and girls: 8.9%) while boys reported more exposure to psychological violence (boys: 21.7% and girls: 18.5%) and any form of violence (boys: 23.7% and girls: 21.7%). The results revealed that girls with functional difficulties (disability) were more exposed to any forms of violence in comparison to disabled boys. Similarly, out-of-school and orphaned girls reported more exposure to any form of violence. On the other hand, boys from households with an individual experiencing depression or mental health problems reported more exposure to any form of violence. Factors associated with both physical and psychological violence were age, school attendance, disability status, living in a household with access to safety nets, a member with mental health problems, positive household consumption, and living in Muslim-majority communities and communities that take advantage of each other were associated only with psychological violence. Risk factors for physical violence only were living in communities that do not have a good relationship with each other and those without mobile network coverage.

**Table 3:** Bivariate association between exposure to physical and psychological violence and various family background characteristics of adolescents in Burkina Faso

Background Characteristics	Physical Violence			Psychological Violence			Any Violence		
	**Male**	**Female**	**All**	**Male**	**Female**	**All**	**Male**	**Female**	**All**
	**%**	**%**	**%, *p* < 0.05**	**%**	**%**	**%, *p* < 0.05**	**%**	**%**	**%, *p* < 0.05**
**Age Group**									
10–14 Years	11.5	11.5	11.5^*^	23.6	19.9	21.8^*^	26.2	23.8	25.0^*^
15–19 Years	4.1	5.2	4.7	18.0	16.0	17.0	19.1	18.2	18.6
**Attended School**									
Yes	11.5	9.5	10.5^*^	25.6	21.3	23.4^*^	28.6	24.5	26.5^*^
No	6.0	8.6	7.2	17.0	14.7	15.9	18.0	17.9	17.9
**Disability Status**									
Yes	28.6	57.1	38.1^*^	35.7	42.9	38.1^*^	42.9	57.1	47.6^*^
No	8.7	8.8	8.8	21.5	18.3	19.9	23.5	21.5	22.5
**Orphanhood Status**									
Not Orphan	9.5	9.2	9.3	21.7	17.6	19.6	24.0	21.1	23.2
Orphan	6.0	9.3	7.6	22.2	24.2	23.2	22.8	26.1	22.5
**Household Size**									
0–6	9.8	9.0	9.4	24.6	17.1	20.6	26.7	20.3	19.2
> 6	8.7	9.3	9.0	20.6	19.1	19.9	22.7	22.4	23.2
**Wealth Quintile**									
First quintile	11.1	9.6	10.3	18.6	16.8	17.7	20.6	20.0	20.3
Second quintile	8.9	8.6	8.7	19.6	18.6	19.1	21.3	21.7	21.5
Third quintile	9.2	6.9	8.1	18.8	19.8	19.8	22.1	23.0	22.5
Fourth quintile	8.6	12.2	10.3	24.6	16.3	20.6	25.9	21.0	23.5
Fifth quintile	7.3	8.9	8.1	26.4	20.8	23.5	28.2	23.0	25.7
**Household has inadequate food**									
Yes	10.5	9.7	10.1^*^	20.9	17.6	19.4	23.5	21.8	22.7
No	7.4	8.6	8.0	22.4	19.4	21.2	23.9	21.7	22.8
**Household Access to Safety Nets**									
Yes	12.1	10.3	11.2^*^	30.3	24.4	27.5^*^	34.5	27.9	31.4^*^
No	7.8	8.8	8.3	18.4	16.4	17.4	19.6	19.6	19,6
**Household has a depressed person**									
Yes	25.5	17.5	21.3^*^	37.3	31.6	34.3^*^	41.2	35.1	38.0^*^
No	8.2	8.5	8.4	21.0	17.7	19.4	23.0	21.0	22.0
**Household has alcohol consumption**									
Yes	3.8	4.0	3.8^*^	16.0	10.3	13.2^*^	18.9	13.1	16.0^*^
No	9.5	9.7	9.6	22.2	19.4	20.8	24.2	22.7	23.4
**Community members take advantage of each other**									
** Yes**	8.8	9.0	9.1	18.6	17.4	18.0^*^	21.0	20.9	20.9^*^
** No**	9.1	9.4	9.0	24.0	19.5	21.8	25.8	22.5	24.2
**Community has Muslim majority**									
Yes	10.3	10.8	10.5^*^	26.7	22.3	24.6*	28.8	26.6	27.7^*^
No	6.9	6.7	6.8	14.1	12.8	13.4	16.1	14.6	15.3
**Community has mobile network**									
Yes	11.4	9.9	10.6^*^	21.3	19.6	19.4	24.2	23.2	23.7
No	4.8	8.1	6.5	22.2	16.8	20.6	23.0	19.5	21.2
**Total**	**8.9**	**9.2**	**9.1**	**21.7**	**18.5**	20.1^*^	**23.7**	**21.7**	**22.7**

In this table, we conducted a Chi-squared test of association

^*^p < 0.05

### Multivariable logistic regression

Tables 4 and 5 show the results from the multivariable logistic regressions examining the association between the child's background characteristics and exposure to violence. Child-level socio-demographic characteristics associated with exposure to any form of violence include age, school attendance, orphanhood, and disability status. Older age was a protective factor for exposure to both physical and psychological violence among both boys and girls. Adolescents aged 15–19 were 30% less likely to be exposed to any form of violence when compared to younger adolescents aged 10–14 years (aOR = 0.70; 95% CI = (0.55–0.89). Furthermore, among boys, the risk of exposure to physical violence within the household was higher among younger adolescent boys (aOR = 0.31; 95% CI = (0.18–0.55). Similarly, adolescent girls' older age, is correlated with a lower risk of exposure to physical violence in the household, however, this reduction is lower when compared to boys (aOR = 0.41; 95% CI = (0.24–0.71). On psychological violence, adolescents aged 15–19 were 25% less likely to be exposed to this form of violence (aOR = 0.75; 95% CI = (0.59–0.96). However, age does not influence exposure to psychological violence among either boys or girls alone.

**Table 4:** Multivariable logistic regression for the association between exposure to psychological and physical violence by background characteristics among boys and girls

Background Characteristics	Physical Violence			Psychological Violence		
	**Model 1**	**Model 2-Male**	**Model 3-Female**	**Model 1**	**Model 2-Male**	**Model 3-Female**
**Sex**	1.02(0.77–1.36)	-	-	0.81(0.64–1.04)	-	-
**Age Group (Ref:10–14)**	0.37^***^(0.26–0.5)	0.31^***^(0.18–0.55)	0.41^***^(0.24–0.71)	0.76^*^(0.59–0.96)	0.70(0.49–1.01)	0.78(0.55- 1.12)
**Attended School**	1.09(0.79–1.50)	1.47(0.93–2.30)	0.79(0.49–1.30)	1.38^**^(1.09–1.73)	1.47*(1.06–2.03)	1.31(0.98–1.77)
**Disabled**	6.52^***^(2.67–15.92)	5.7^**^(1.71–19.10)	11.11^**^(2.22- 55.68)	2.46*(1.12–5.36)	2.56(0.91–7.19)	2.51(0.59–10.72)
**Orphaned Child**	0.82(0.57–1.17)	0.63(0.31- 1.27)	1.06(0.58–1.93)	1.36^*^(1.07–1.73)	1.06(0.74–1.55)	1.78^**^(1.20–2.66)
**Wealth Quintile (Ref: First quintile)**						
Second quintile	0.90(0.58–1.41)	0.84(0.48–1.47)	0.97(0.49–1.91)	1.12(0.79–1.58)	1.10(0.74–1.65)	1.13(0.65–1.95)
Third quintile	0.83(0.54–1.27)	0.82(0.46–1.45)	0.81(0.42–1.57)	1.10(0.76–1.60)	0.93(0.58–1.51)	1.34(0.78–2.30)
Fourth quintile	0.97(0.66–1.43)	0.74(0.40–1.33)	1.25(0.68–2.28)	1.13(0.76–1.68)	1.40(0.78–2.49)	0.84(0.49–1.42)
Fifth quintile	0.75(0.45–1.28)	0.63(0.37–1.10)	0.89(0.39–2.04)	1.47^*^(1.01—2.14)	1.74^*^(1.08–2.81)	1.21(0.73–2.02)
**Household Size (Ref < 0–6)**	0.91(0.63–1.32)	0.89(0.53–1.48)	0.92(0.57–1.48)	0.95(0.70–1.29)	0.76(0.52–1.12)	1.17(0.78–1.75)
**Household has inadequate food**	1.25(0.81–1.95)	1.49(0.83–2.70)	1.09(0.69–1.71)	0.91(0.66–1.27)	0.97(0.64–1.45)	0.84(0.54–1.30)
**Household has Access to Safety Nets**	1.380.88–2.17)	1.67(0.95- 2.91)	1.20(0.70–2.03)	1.68^**^(1.21–2.36)	1.86^**^(1.23–2.83)	1.61^*^(1.08–2.40)
**Household has a depressed person**	2.65^***^(1.67–4.23)	3.44^***^(1.96–6.03)	1.96(0.83–4.46)	2.13^**^(1.22–3.70)	2.12^*^(1.10–4.08)	2.04(0.96- 4.36)
**Household has alcohol consumption**	0.36^**^(0.18–0.72)	0.41(0.14–1.14)	0.39(0.12–1.22)	0.62^*^(0.39–1.00)	0.714(0.39–1.26)	0.54(0.29–1.05)
**Community members take advantage of each other**	1.06(0.76–1.50)	1.04(0.57–1.92)	1.10(0.74–1.63)	0.77(0.59–1.01)	0.68^*^(0.47–1.00)	0.88(0.63–1.24)
**Community has Muslim majority**	1.50(0.83–2.7)	1.04(0.70–2.79)	1.63(0.87–3.02)	1.90^***^(1.44–2.50)	2.00^***^(1.35–2.95)	1.86^***^(1.29–2.67)
**Community has mobile network**	1.65(0.90–3.04)	2.26^*^(1.03–4.97)	1.30(0.70–2.41)	1.02(0.78–1.34)	0.84(0.58–1.21)	1.24(0.88–1.77)
**Pseudo R2**	**0.07**	**0.11**	**0.06**	**0.05**	**0.07**	**0.05**
**Goodness of fit**	**0.28**	**0.43**	**0.46**	**0.16**	**0.04**	**0.45**

*OR* adjusted odds ratio

^***^ p < 0.001

^**^ p < 0.01

^*^ p < 0.05

**Table 5:** Multivariable logistic regression for the association between exposure to any form of violence by background characteristics among boys and girls

Background Characteristics	Any Violence		
	**Model 1**	**Model 2-Boys**	**Model 3-Girls**
	**OR (95% CI)**	**OR (95% CI)**	**OR (95% CI)**
**Sex**	0.90(0.72–1.13)	-	-
**Age (Ref:10–14)**	0.70^**^(0.55–0.89)	0.66^*^(0.47–0.93)	0.72(0.50–1.05)
**Attended School**	1.37^*^(1.08–1.73)	1.55^*^(1.10–2.18)	1.21(0.90–1.63)
**Disabled**	3.17^**^(1.41–7.14)	3.09^*^(1.12–8.54)	3.81(0.90–16.07)
**Orphaned Child**	1.20(0.94–1.55)	0.95(0.65–1.40)	1.54^*^(1.08–2.19)
**Wealth Quintile (Ref:** First quintile)			
Second quintile	1.10(0.80–1.53)	1.09(0.75–1.58)	1.11(0.68–1.84)
Third quintile	1.15(0.83–1.60)	1.03(0.67–1.59)	1.30(0.78–2.17)
Fourth quintile	1.13(0.80–1.60)	1.31(0.77–2.24)	0.93(0.58–1.50)
Fifth quintile	1.38(0.95–1.99)	1.70^*^(1.06–2.72)	1.11(0.67–1.84)
**Household Size (Ref < 0–6)**	0.95(0.71–1.27)	0.75(0.50 -1.13)	1.15(0.82–1.61)
**Household has inadequate food**	1.02(0.74–1.40)	1.05(0.71–1.58)	0.97(0.67–1.42)
**Household has Access to Safety Nets**	1.77^***^(1.29–2.43)	2.13^***^(1.43–3.16)	1.56^*^(1.09–2.23)
**Household has a depressed person**	2.08^*^(1.19–3.64)	2.19^*^(1.14–4.20)	1.88(0.86–4.10)
**Household with alcohol consumption**	0.66(0.43–1.03)	0.74(0.43–1.27)	0.59(0.32–1.08)
**Community members take advantage of each other**	0.82(0.63–1.07)	0.73(0.48–1.10)	0.92(0.71–1.21)
**Community has Muslim majority**	1.93^***^(1.39–2.67)	1.87^**^(1.21–2.87)	2.04^***^(1.44- 2.88)
**Community has mobile network**	1.10(0.83–1.45)	0.93(0.63–1.38)	1.26(0.90–1.77)
**Pseudo R2**	0.06	0.07	0.05
**Goodness of fit**	0.23	0.07	0.50

*OR* adjusted odds ratio

^***^ p < 0.001

^**^ p < 0.01

^*^ p < 0.05

Attending school was a significant risk factor for exposure to psychological violence and not physical violence among all children and boys. Children attending school were 1.4 times more likely to be exposed to psychological violence when compared to their peers who were out of school (aOR = 1.38; 95% CI = (1.09–1.73). Additionally, children experiencing functional disabilities were associated with a higher risk of exposure to all forms of violence. Adolescents with functional disabilities (OR 3.17, 95% CI 1.41–7.14) had statistically significantly lower odds of experiencing any form of violence. Disabled children were 6.5 times more likely to be exposed to physical violence, with girls having more chances of exposure to physical violence than boys (aOR = 5.7; 95% CI = (1.71–19.10; aOR = 11.1; 95% CI = (2.22–55.68)). Orphanhood was a significant factor in exposure to psychological violence only especially among girls. Compared with girls with both parents, orphaned girls were 1.8 times more likely to be exposed to psychological violence (aOR = 1.78; 95% CI = (1.20–2.66).

Living in a household with access to safety nets and living in the same household with a depressed individual were important household-level risk factors for exposure to violence among the children in the sample. Among all children in the sample, the odds of exposure to any form of violence were higher among those living with a person experiencing mental health problems (aOR = 2.08; 95% CI = (1.19–3.64). Similarly, boys who reported having an individual in the household who has mental health problems were between 2.1–3.5 times more likely to be exposed to both physical and psychological violence. Living with a family member with mental health problems was not a significant risk factor among girls for either form of violence. Children living in households receiving social safety nets faced a higher risk of exposure to violence. The odds of exposure to any form of violence were higher among children from households receiving safety nets when compared to their counterparts (aOR = 1.77; 95% CI = (1.29–2.43).

While not a risk factor for physical violence, living in households receiving any form of safety net was a risk factor for exposure to psychological violence among boys and girls. For instance, among boys, those living in households receiving any form of safety net were 1.9 times more likely to be exposed to violence when compared to their peers in households not receiving safety nets (aOR = 1.86; 95% CI = (1.23–2.83). Among girls, only the odds of exposure to psychological violence were 1.6 times higher among those who live in households receiving safety nets (aOR = 1.61; 95% CI = (1.08–2.40).

The community social environment was an important risk factor for exposure to violence, especially among girls. Living in Muslim-majority communities was a significant risk factor for exposure to any form of violence and psychological violence for both boys and girls while remaining significant for physical violence. Boys living in Muslim-majority communities were twice more likely to be exposed to psychological violence (aOR = 2.00; 95% CI = (1.35–2.95). Similarly, girls residing in the same communities were 1.8 times more likely to be exposed to psychological violence when compared to girls living in non-Muslim majority communities (aOR = 1.86; 95% CI = (1.29–2.67).

A child’s sex was not a risk factor for either psychological or physical violence. Similarly, at the household level, household size, wealth, household food status food and positive alcohol consumption were not associated with children’s exposure to any form of violence. Furthermore, living in a community without mobile coverage and among members who take advantage of each other was not associated with either form of violence.

## Discussion

This study examined the risk and protective factors associated with exposure to household violence among adolescents in four regions of Burkina Faso. Similar to previous studies [29], our findings show that more adolescents were exposed to psychological violence. Increases in exposure to psychological violence among adolescents may be linked to the increase in the insecurity levels in the regions in the Sahel belt [27]. In recent years, climate shocks and political insecurity in the Sahel belt have taken hold, thereby adding more misery to an already precarious condition. Additionally, in affected areas, educational activities have been disrupted resulting in children staying home for too long, thereby even facing greater risks of exposure to violence [36, 37]. Furthermore, violence at home in Burkina Faso is more widespread as shown by a national survey of violence among children [30].

The study finds that the sex of the adolescent child was not statistically associated with exposure to violence, similar to what other studies in sub-Saharan Africa [38]. The interactions children and adolescents have within families, schools, and communities foster healthy growth and development by providing a child with love, emotional support, and opportunities for learning and exploration [11]. Therefore, understanding the contexts where adolescents are likely to be exposed to physical and psychological violence both at home and in the community is critical to safeguarding adolescents’ needs and to better-preventing violence. In this study, we found that age protected children and adolescents from exposure to violence, with older age associated with a lower risk of exposure to violence. This is consistent with a recent report on children in Burkina Faso that showed that the risk of violence among children aged 0–11 was nearly double that for those aged 12–17 [39]. The explanation for this could be that younger children may face violent child discipline both at home and within schools when compared to older adolescents who are out of school. In Burkina Faso, corporal punishment is still to be outlawed in the home, alternative care settings, day-care, schools, and penal institutions thereby putting children at greater risk [40]. Since corporal punishment is not prohibited in schools, as shown in our study, school attendance was positively correlated with exposure to psychological violence. Adolescents attending school might be exposed to violence from their teachers and peers, as shown by previous studies in Burkina Faso which show that apart from home, schools were the second most common place for violence against children [30]. Orphaned children were more likely than non-orphans to be exposed to household violence. Disability among children and adolescents was a risk factor for exposure to both physical and physical violence. Similarly, orphaned children and adolescents especially girls were more likely to be exposed to violence in the household. This is consistent with findings from previous research conducted in sub-Saharan Africa on violence against children [11, 14, 15].

A safe and secure home environment is conducive to child growth and development. Our study finds that adolescent in households receiving social safety nets were more likely to be exposed to violence. The explanation for this could be that the most vulnerable households received safety nets, and not necessarily that safety nets cause violence. In this study, we also found out that living with a household member with mental health problems is associated with a greater likelihood of exposure to violence among children and adolescents. Mental illness may cause a variety of psychosocial problems such as decreased quality of life of the patient’s family members as well as increased stress and anxiety [41]. Apart from exposing children and adolescents to violence, mental health challenges such as aggression and suicide ideation can be a direct consequence of violence against children [22–25]. Our study also finds adolescents living in Muslim-majority communities were at a heightened risk of exposure to violence. Protective communities are essential for the prevention of violence against children, especially in countries such as Burkina Faso which has witnessed increased ethnic and religious conflicts [42]. Religion and ethnicity have been identified with risks of violence against children and adolescents in Burkina Faso [28, 43, 44].

This study highlights the crucial factors associated with exposure to both physical and psychological violence. It should be important to note that psychological violence is still not well understood in these communities which may have resulted in underreporting. However, this study presents evidence of the risk factors within the household and at the community level. The study had several limitations. First, the sample size is not large enough to provide nationally representative estimates. The study was based on a cross-sectional survey which could only allow us to show factors associated with exposure to violence, and not establish causal relationships. The study might have encountered issues of recall bias, leading to under and over-reporting of exposure to violence and other variables. Children and adolescents exposed to violence from their caregivers at home could have been more reticent to fully report the experiences, especially since the interviews were conducted at home. 

Further research that utilizes longitudinal designs is needed to monitor the prevalence and predictors of violence in poor populations beset by multiple crises such as conflict and climate change. More qualitative evidence might help explain the role of social norms in adolescents' exposure to violence. An examination of resilience among children and adolescents and how it influences the impact of exposure to violence in these contexts is another area of future research [45]. In addition, studies can also determine how long-term exposure to violence during childhood and adolescence in such contexts affects later life well-being, personality traits and behaviours including participation in armed conflict.

## Conclusion

Several individual, family and community-level factors are associated with exposure to violence among adolescents in Burkina Faso. They include age, schooling, disability, orphanhood, living with a person with poor mental health, household vulnerability and living in Muslim-majority communities. This study’s findings highlight the need for implementing and strengthening programmes and interventions aimed at reducing violence against children and adolescents at home and in schools. Special attention should be given to vulnerable children especially orphans and those with disabilities who might not be able to report violence. Furthermore, prevention and response programmes can sensitize public service personnel (social work and case management, education, health care and law enforcement) and provide disability-focused service centres [46]; while poverty alleviation programmes (e.g. cash transfers) can establish reporting and referral mechanisms that link vulnerable children with case management services [47]. The ongoing conflict in Burkina Faso puts children and adolescents at even greater risk of violence, especially in the worst affected communities. Additionally, reducing violence against children and adolescents remains critical, especially in the context of socio-economic vulnerabilities caused by COVID-19, climate change and conflict-induced food price crises.

## Supplementary Information

**Additional file 1:** **Supplementary Table 1.** Sample characteristics of study population., (*N*=2222). **Supplementary Table 2.** Goodness-of-fit test Results. **Supplementary Table 3.** Multicollinearity Tests for All Children Model. **Supplementary Table 4.** Multicollinearity Tests for Males Model. **Supplementary Table 5.** Multicollinearity Tests for Female Model.

## Data Availability

The datasets used and/or analysed during the current study are available from the corresponding author on reasonable request.

## Footnotes

1 This question was taken from the adolescent module of the “Study to profile child poverty and vulnerability in the East, North and Centre region in Burkina Faso” which used Multidimensional Overlapping Deprivation Analysis (MODA) to measure exposure to violence for adolescents and which was pretested in the local context. As part of our study, the enumerators were trained to provide examples of forms of violence, to contextualise the meaning of violence and to differentiate physical and psychological violence from other forms of violence. However, given that the meaning of violence is relative, the question used in our study captures the adolescents’ perceptions of exposure to violence, which depends on their socio-cultural context, as well as their individual life experience.

2 The asset index was calculated based on the type of toilet in the home, presence or absence of household electricity, ownership of various household items, ownership of means of transportation, source of energy used for cooking, number of rooms in the household used for sleeping, type of flooring and roofing materials, type of material used for walls, and source of drinking water.
