# Supplementary material for: Risk factors of adolescent exposure to violence in Burkina Faso
Source: BMC Public Health. 2022 Dec 21;22:2405. doi: 10.1186/s12889-022-14854-7 (PMC9771600; doi:10.1186/s12889-022-14854-7)
Supplement: Supplementary file 1 — Additional file 1: Supplementary Table 1. Sample characteristics of study population., (N=2222). Supplementary Table 2. Goodness-of-fit test Results. Supplementary Table 3. Multicollinearity Tests for All Children Model. Supplementary Table 4. Multicollinearity Tests for Males Model. Supplementary Table 5. Multicollinearity Tests for Female Model. [file 12889_2022_14854_MOESM1_ESM.docx]

1. **Supplementary Tables**

**Supplementary Table 1: Sample characteristics of study population., (N=2222)**

| **Variable** | **Male** | **Female** | **Total** | **Number** |
| --- | --- | --- | --- | --- |
| **Household has inadequate food** |  |  |  |  |
| Yes | 87.1 | 87.5 | 87.3 | 1940 |
| No | 12.9 | 12.5 | 12.7 | 282 |
| **Household Access to Safety Nets** |  |  |  |  |
| Yes | 27.5 | 25.6 | 25.6 | 590 |
| No | 72.5 | 74.4 | 74.4 | 1632 |
| **Household has a depressed person** |  |  |  |  |
| Yes | 4.6 | 5.2 | 4.9 | 108 |
| No | 95.4 | 94.8 | 95.1 | 2092 |
| **Household has positive alcohol consumption** |  |  |  |  |
| Yes | 9.5 | 9.7 | 9.6 | 213 |
| No | 9.5 | 90.3 | 90.4 | 2009 |
| **Community members take advantage of each other** |  |  |  |  |
| **Yes** | 43.8 | 46.5 | 45.1 | 1003 |
| **No** | 56.2 | 53.6 | 54.9 | 1219 |
| **Community has Muslim majority** |  |  |  |  |
| Yes | 59.9 | 59.6 | 59.8 | 1324 |
| No | 40.1 | 40.4 | 40.2 | 894 |
| **Community has mobile network** |  |  |  |  |
| Yes | 63.0 | 60.6 | 61.8 | 1373 |
| No | 37.0 | 39.4 | 38.2 | 849 |
| **Region** |  |  |  |  |
| Boucle Du Mouhoun | 23.0 | 18.8 | 20.9 | 464 |
| East | 16.9 | 19.7 | 18.3 | 408 |
| North | 30.6 | 33.7 | 32.1 | 714 |
| Centre-North | 29.5 | 27.9 | 28.7 | 638 |
| **Total** | **1118(50.3)** | **1104(49.7)** | **2222(100.0)** | **2222** |

**Supplementary Table 2: Goodness-of-fit test Results**

| **Model** | **Number of observations** | **Number of covariate patterns** | | **Pearson chi square** | **Prob > chi2** |
| --- | --- | --- | --- | --- | --- |
| **Physical violence – All Children** | 2184 | | 1486 | 1499.87 | 0.2755 |
| **Physical violence - Male** | 1103 | | 746 | 734.68 | 0.4341 |
| **Physical violence - Female** | 1081 | | 740 | 726.21 | 0.4594 |
| **Psychological violence-All Children** | 2184 | | 1486 | 1522.31 | 0.1581 |
| **Psychological violence-Male** | 1103 | | 746 | 796.77 | 0.0409 |
| **Psychological violence-Female** | 1081 | | 740 | 727.08 | 0.4504 |
| **Any Violence -All Children** | 2184 | | 1486 | 1508.44 | 0.2261 |
| **Any Violence-Male** | 1103 | | 746 | 785.45 | 0.0723 |
| **Any Violence-Female** | 1081 | | 740 | 722.08 | 0.5026 |

**Supplementary Table 3. Multicollinearity Tests for All Children Model**

| **vif, uncentered** |  |  |
| --- | --- | --- |
| **Variable** | **VIF** | **1/VIF** |
| sex1 | 6.05 | 0.165323 |
| 2.age_group | 1.59 | 0.628327 |
| att_school | 2.42 | 0.413522 |
| disability | 1.01 | 0.985559 |
| 1.orphanhood | 1.19 | 0.838105 |
| 2.hhsize_cat | 3.51 | 0.285019 |
| wquintile |  |  |
| 1 | 1.90 | 0.527093 |
| 2 | 1.91 | 0.522353 |
| 3 | 1.91 | 0.523948 |
| 4 | 2.00 | 0.499575 |
| hh_situati~s | 2.05 | 0.487489 |
| safetynets | 1.42 | 0.704092 |
| take_advan~e | 1.80 | 0.555809 |
| depression | 1.07 | 0.937279 |
| alcohol | 1.13 | 0.883276 |
| musmaj1 | 2.44 | 0.409359 |
| fullmobnet1 | 2.62 | 0.381794 |
|  |  |  |
| Mean VIF | 2.12 |  |

**Supplementary Table 4. Multicollinearity Tests for Males Model**

| Variable | VIF | 1/VIF |
| --- | --- | --- |
| 2.age_group | 1.53 | 0.652012 |
| att_school | 2.28 | 0.438440 |
| disability | 1.02 | 0.982750 |
| 1.orphanhood | 1.19 | 0.838357 |
| 2.hhsize_cat | 3.59 | 0.278728 |
| wquintile |  |  |
| 1 | 1.82 | 0.550380 |
| 2 | 1.89 | 0.528385 |
| 3 | 1.82 | 0.548367 |
| 4 | 1.93 | 0.517206 |
| hh_situati~s | 2.01 | 0.498045 |
| safetynets | 1.48 | 0.677879 |
| take_advan~e | 1.69 | 0.591102 |
| depression | 1.07 | 0.932399 |
| alcohol | 1.14 | 0.880032 |
| musmaj1 | 2.38 | 0.420937 |
| fullmobnet1 | 2.60 | 0.384163 |
| Mean VIF | 1.84 |  |

**Supplementary Table 5. Multicollinearity Tests for Female Model**

| Variable | VIF | 1/VIF |
| --- | --- | --- |
| 2.age_group | 1.59 | 0.628073 |
| att_school | 2.45 | 0.408744 |
| disability | 1.03 | 0.974955 |
| 1.orphanhood | 1.18 | 0.849656 |
| 2.hhsize_cat | 3.14 | 0.318217 |
| wquintile |  |  |
| 1 | 1.80 | 0.556969 |
| 2 | 1.75 | 0.571842 |
| 3 | 1.81 | 0.553926 |
| 4 | 1.89 | 0.527888 |
| hh_situati~s | 2.02 | 0.495566 |
| safetynets | 1.38 | 0.725172 |
| take_advan~e | 1.80 | 0.555005 |
| depression | 1.07 | 0.931698 |
| alcohol | 1.13 | 0.887349 |
| musmaj1 | 2.37 | 0.421550 |
| fullmobnet1 | 2.51 | 0.397882 |
| Mean VIF | 1.81 |  |
|  |  |  |
